# Supplementary material for: Differentially expressed fusogens specify myocyte states to drive myogenesis
Source: Development. 2025 Sep 30;152(19):dev204771. doi: 10.1242/dev.204771 (PMC12517348; doi:10.1242/dev.204771)
Supplement: Supplementary information [file develop-152-204771-s1.pdf]

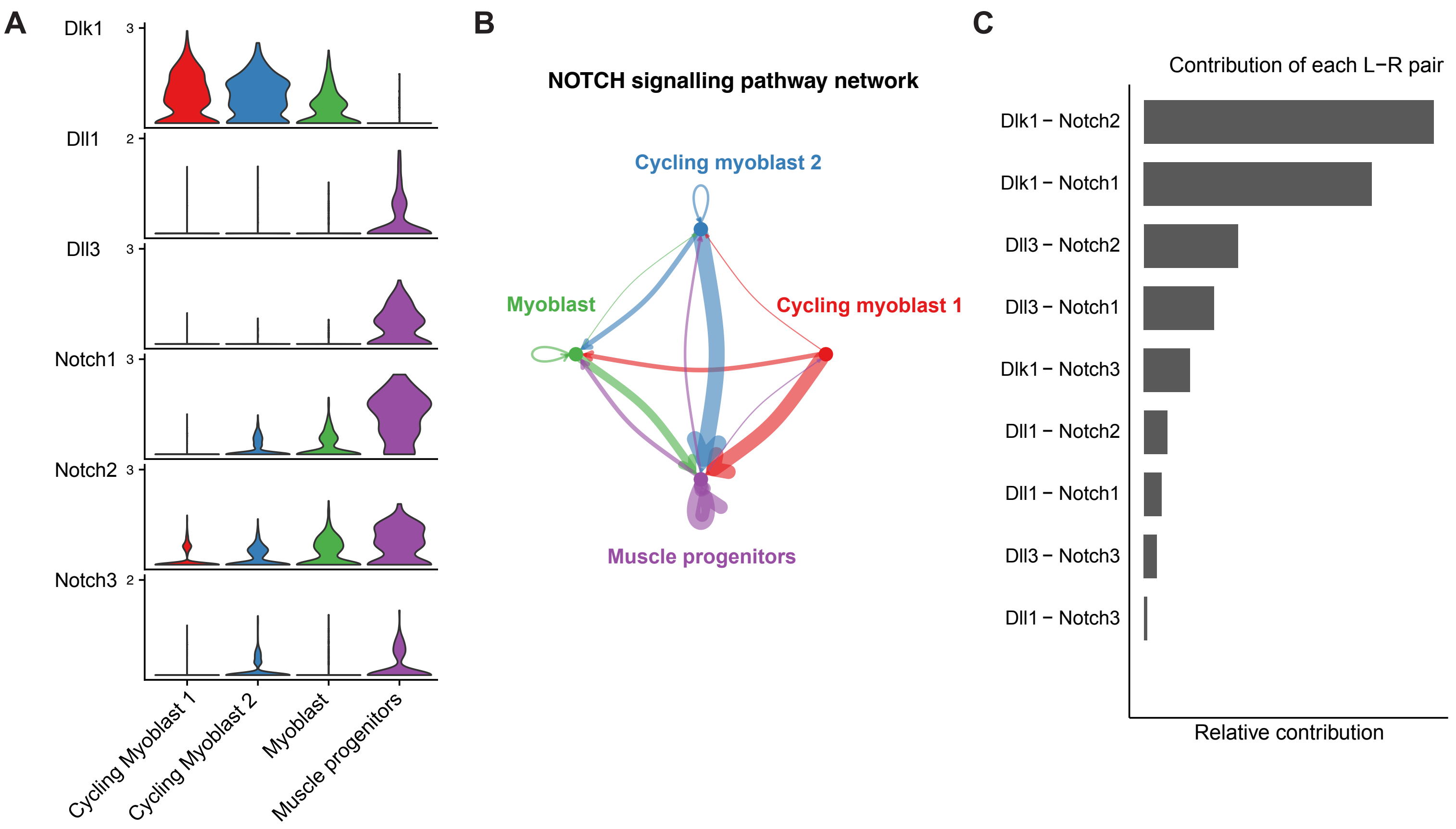

Fig. S1. The E9.5 progenitor populations likely communicate through the Notch signaling pathway.

(A) Violin plot showing the expression levels of the Notch signaling members in E9.5 muscle progenitors. (B) CellChat analysis showing the predicted communication direction between the progenitor populations *via* the Notch signaling pathway. The arrow line thickness represent confidence in the communication. (C) Bar graph showing the relative contribution of each receptor-ligand pair in the predicted communication between the population under the Notch pathway.

Development • Supplementary information

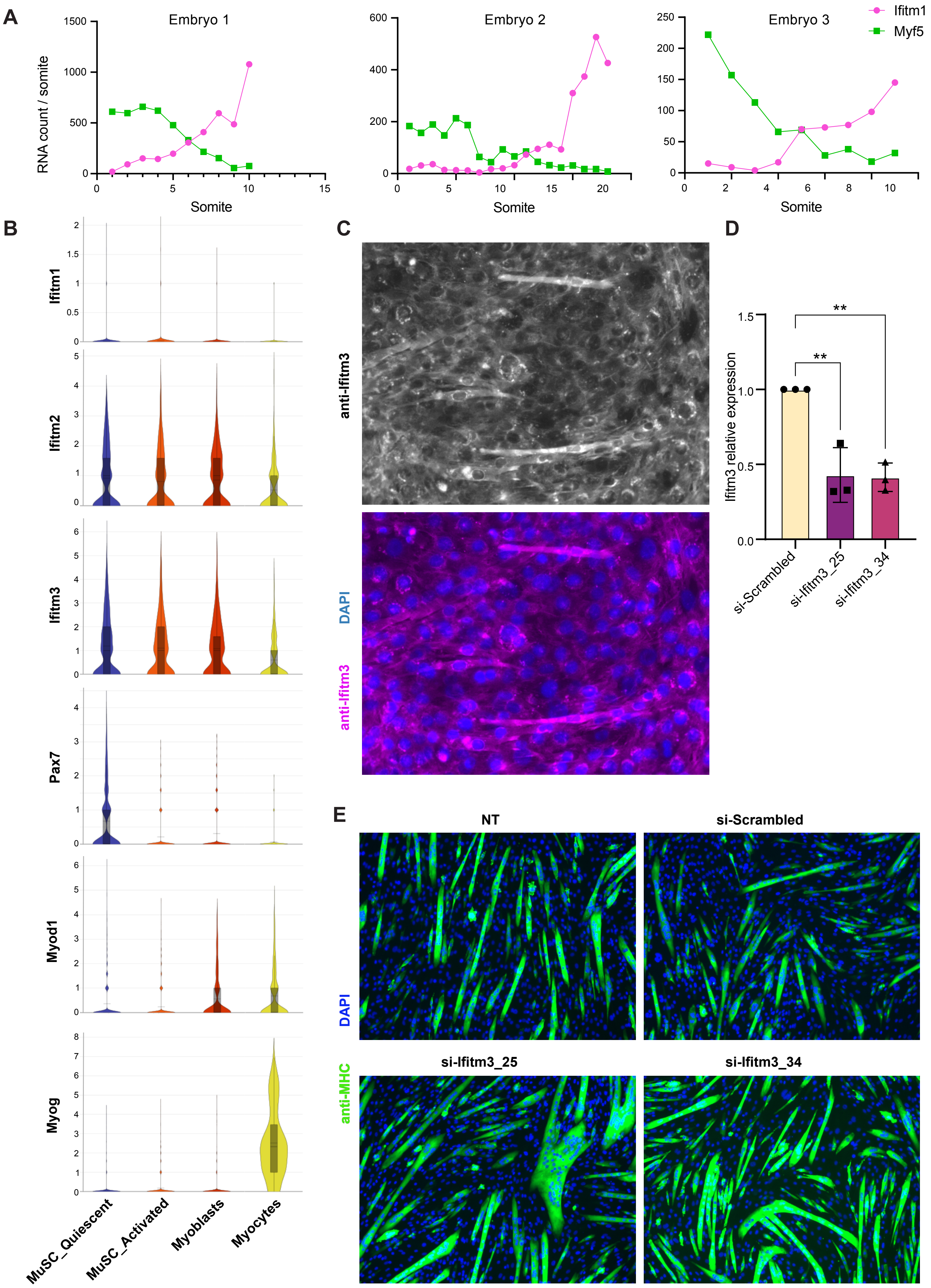

Development • Supplementary information

Fig. S2. *Ifitm* genes expression in adult muscle cells.

(A) Quantification of *Myf5* and *Ifitm1* RNA counts per somite using the fluorescent *in situ* hybridization in E9.5 embryo sections. Somite number 1 represent the first visible somite in the anterior part of the embryo section. (B) *Ifitm2* and *Ifitm3* expression is higher in Quiescent and activated MuSCS and, myoblasts compared to Myocytes. *Ifitm1* gene is not expressed by adult myogenic cells. Myogenic and myofiber cells were selected from the scMuscle Atlas and dimensionally reduced with PHATE. The PHATE algorithm separated cells along the differentiation axis to visualize gene expression. Analyzed data from (McKellar et al., 2020). (C) Immunofluorescence showing the expression of Ifitm3 in C2C12 differentiated for 24h. (D) qPCR analysis of the *Ifitm3* expression levels in C2C12 transfected with siRNA targeting *Ifitm3*. n=3 independent experiments. The $2^{-\Delta\Delta CT}$ method was used to calculate the relative fold change which was normalized against Gapdh expression. For the statistics, the ordinary one-way ANOVA-multiple comparisons was performed between the control and the treated conditions. Data represents mean ± SD. ns: non-significant. *<0.05, **<0.01, ***<0.001. (E) Immunofluorescence against MHC in Ifitm3 depleted C2C12.

Development • Supplementary information

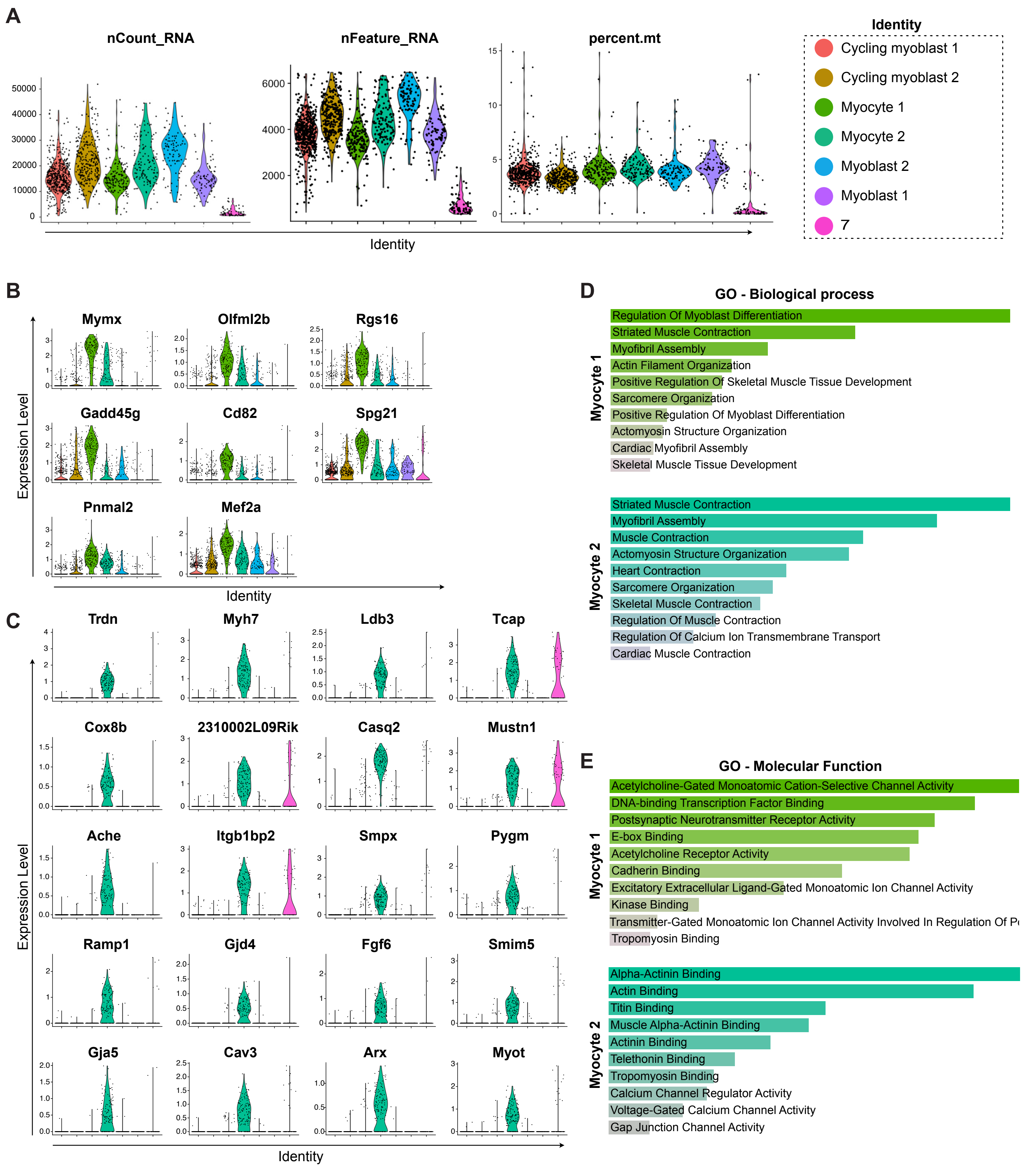

Fig. S3. Mc1 and Mc2 marker analysis shows Mc2 are more mature than Mc1.

(A) Violin plots of quality control counts: RNA count, feature/gene count, mitochondrial genes count. (B) Violin plot of Myocyte 1 (Mc1) marker genes. (C) Violin plot of Myocyte 2 (Mc2) marker genes. (D-E) Gene ontology analysis showing biological process (D) and molecular function (E) for Mc1 and Mc2 populations.

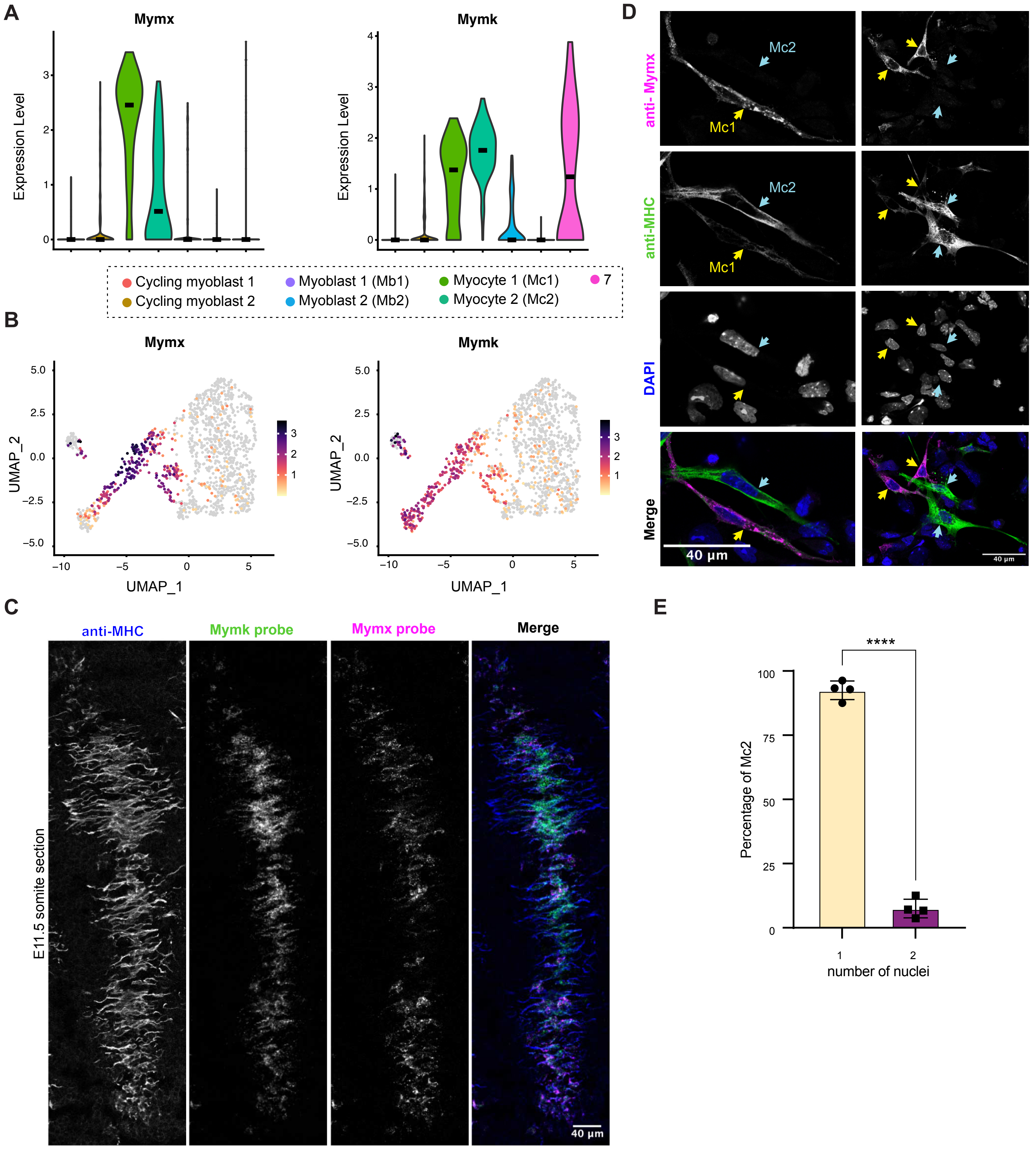

Fig. S4. Mc1 and Mc2 are mononucleated.

(A) Violin plots and Feature plots (B) showing the expression levels of Mymx and Mymk in E11.5 myogenic populations. (C) Fluorescent *in situ* hybridization (same biological samples from Fig. 3G, shown here as single channels) showing the specificity of Mymk (green) and Mymx (magenta) staining in a whole somite section at E11.5. MHC (blue) is stained with MF20 antibody. (D) Immunostaining with anti-MHC and anti-Mymx on *ex vivo* dissociated somite cells from a E11.5 embryo. Mononucleated Mc1 and Mc2 were observed in at least 3 independent embryo preparations. (E) Quantification of the percentage of mono- and bi-nucleated Mc2 cells in *ex vivo* cultures of dissociated somite cells from E11.5 embryos. Unpaired t test was performed. Each point on the graph represents an independent embryo (n=4). Data represents mean ± SD and p-value ***<0.001.

Development • Supplementary information

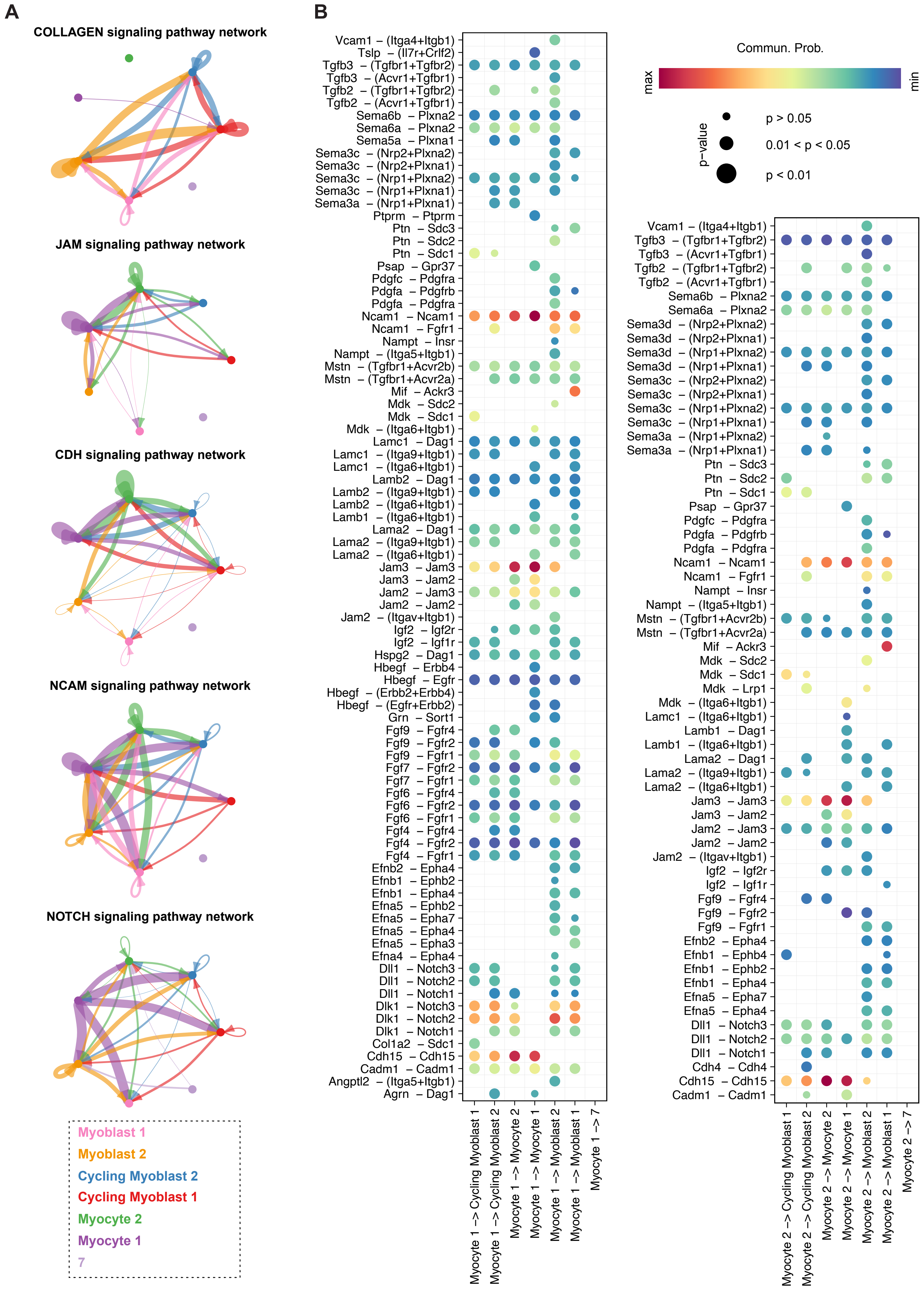

Fig. S5. Mc1 and Mc2 likely interact through adhesion molecules.

(A) CellChat analysis showing the predicted communication direction between the progenitor populations in E11.5 embryos. The arrow line thickness ràepresent confidence in the communication. (B) Dot plot showing the receptor-ligand pair implicated in the communications between the Mc1 versus the rest of the populations and Mc2 versus the rest of the populations.

Development • Supplementary information

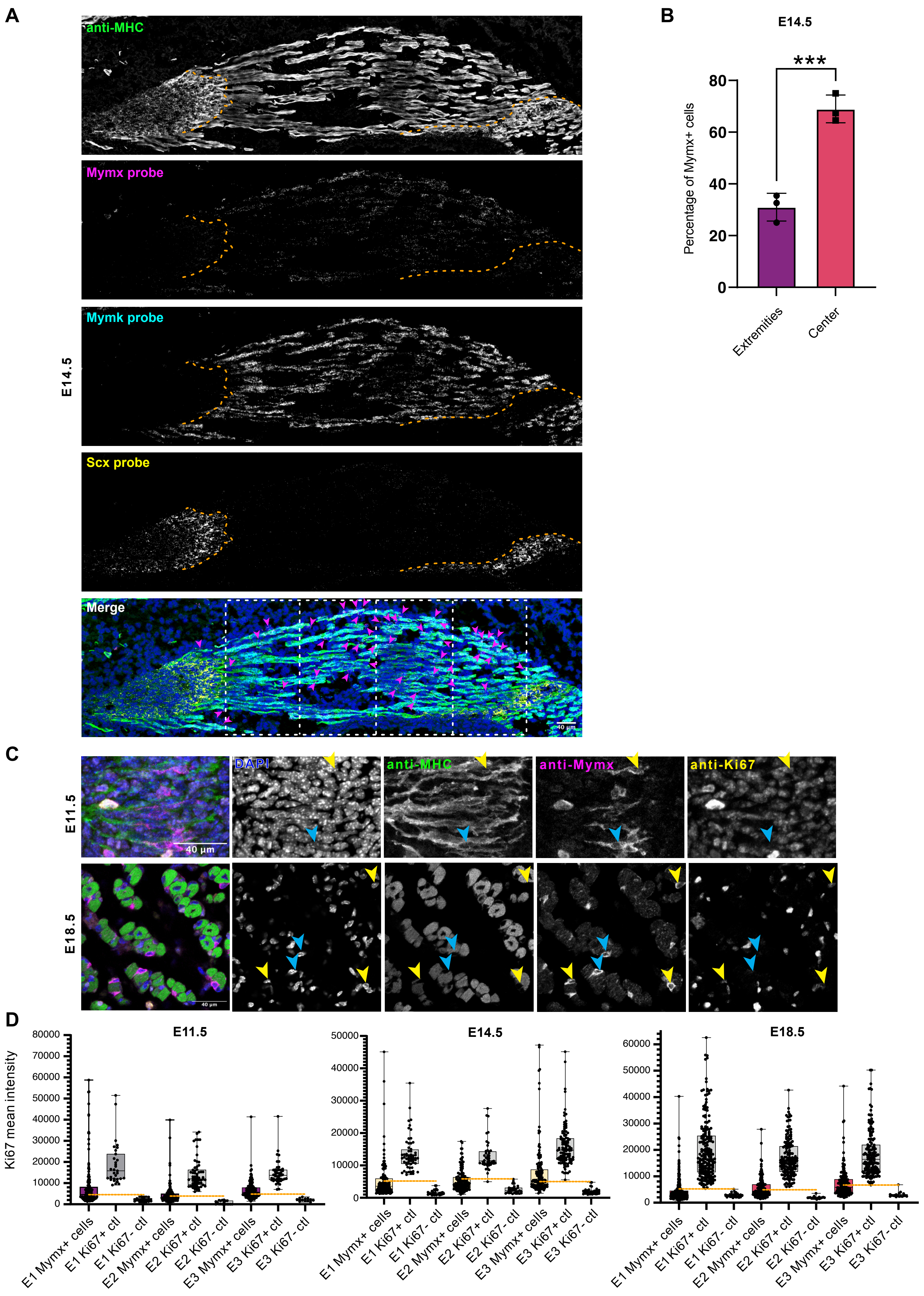

Development • Supplementary information

Fig. S6. The Myocyte 1 are proliferative and positioned in the middle section of the muscle.

(A) Immunostaining against MHC and *in situ* hybridization against Mymx, Mymk and Scx (tendon marker) in limb muscle sections of E14.5 embryos. (B) Quantification of Mymx positive cells position in the E14.5 limb muscle. The muscle was divided into 4 equal parts and Mymx positive cells were counted in each square. The counts in the squares at the extremities were added together and the same was done for the center squares. Each point on the graph represents an independent embryo (n=3). Welch's t test was performed. Data represents mean ± SD and p-value ***<0.001. (C) Immunostaining against MHC, MYMK and Ki67 in sections from embryos aged E11.5 and E18.5. (D) Quantification detail for Ki67 immunostaining in E11.5, E14.5 and E18.5. Mean fluorescence intensity was measured for nuclei (identified by tracing around DAPI staining) in the Ki67 channel. To set a threshold (orange dotted line), internal positive and negative controls were quantified. Finaly, the Ki67 signal was measured for MYMX+ cell nuclei. E1, E2 and E3 represent the separate embryos for each stage.

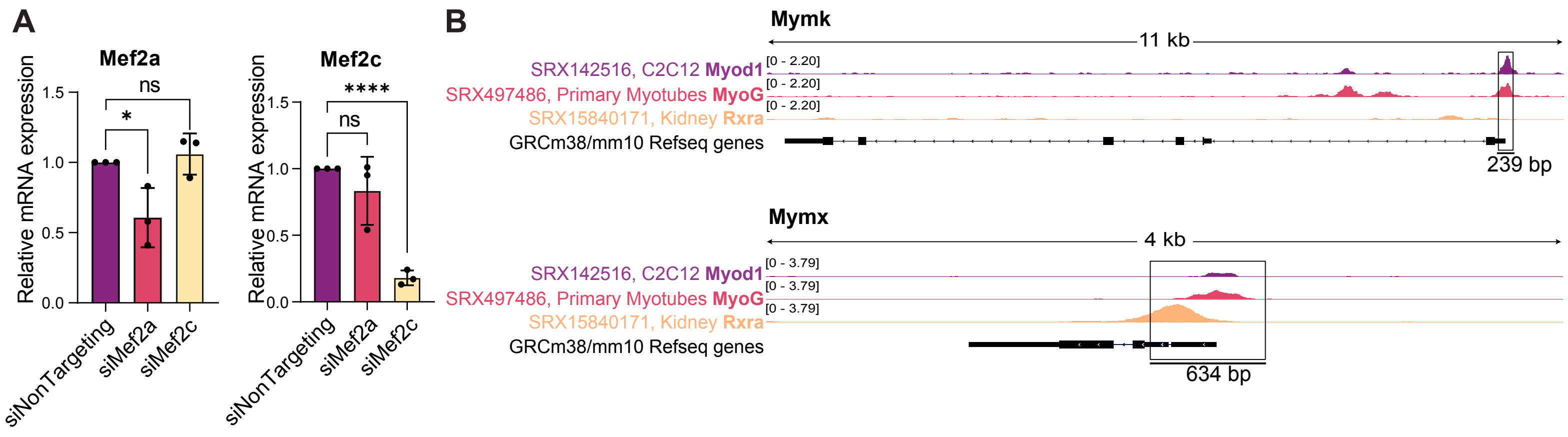

Fig. S7. Validation of Mef2 Knockdown and graph of publicly available ChIP data.

(A) qPCR analysis of *Mef2a*, *Mef2c* expression levels in C2C12 transfected with siRNA targeting *Mef2a* and *Mef2c*. n=3 independent experiments. The $2^{-\Delta\Delta CT}$ method was used to calculate the relative fold change which was normalized against β-actin expression. Each dot represents an independent experiment (n=3). The ordinary one-way ANOVA-multiple comparisons was performed between the control and the treated conditions. Data represents mean ± SD. ns: non-significant. *<0.05, **<0.01, ***<0.001. (B) Chromatin immunoprecipitation and sequencing analysis of publicly available datasets for MYOD1 in C2C12, MyoG in primary myotubes and RXRα in Kidney cells. Regions containing *Mymk* and *Mymx* are shown. MYOD1 and MYOG peaks align on the *Mymk* and *Mymx* promoters respectively. A RXRα peak is present in the first *Mymx* intron. The underline represents the cloned genomic region.

Table S1. Mean percentage of gene expression in the myoblast populations 1 and 2. Calculated from the integrated dataset presented in Fig. 1.

	E9.5	E11.5
Pax3	76.1	59.9
Pax7	8.7	43.5
Myf5	58.6	47.1

Table S2. List of differentially expressed genes (DEGs) of the muscle populations in E11.5 embryos.

The markers of the clusters in Fig. 3 were found using the FindAllMarkers() function of the Seurat R package. Clusters are numbered as follows: Cluster 0 = Cycling myoblast 1, Cluster 1 = Cycling myoblast 2, Cluster 2 = Myocyte 1, Cluster 3 = Myocyte 2, Cluster 4 = Myoblast 2, Cluster 5 = Myoblast 1, Cluster 6 = Technical artifact cluster (re-named cluster 7).

Available for download at
https://journals.biologists.com/dev/article-lookup/doi/10.1242/dev.204771#supplementary-data

Development • Supplementary information

Table S3. Oligonucleotide primers.

Name	Sequence	Purpose
mIfimt3-F	TGCCTACTCCGTGAAGTCTA	Quantitative PCR
mIfitm3-R	GTGTGAAGGTTTTGAGCGTT	Quantitative PCR
Mymk_prom_fw	CATCATGGTACCTGGGGAGAAG<br>GTGGTAAAGGCC	Cloning
Mymk_prom_rev	GATATCCTCGAGGAGAGAGAGAGA<br>GAGAGAGAGAGAGAGAGAGAGAATAT	Cloning
Mymx_prom_fw	CATCATGGTACCCACTCACTGTTA<br>TCACAGATGGGAAGGGA	Cloning
Mymx_prom3_rev	GATATCCTCGAGAACACAGCTTAG<br>CTTCATCTGAGGTGTCAC	Cloning
Myomaker_fw	ATCGCTACCAAGAGGCGTT	Quantitative PCR
Myomaker_rev	CACAGCACAGACAAACCAGG	Quantitative PCR
Myomixer_fw	CTGAGCTCCCAAGACATGAG	Quantitative PCR
Myomixer_rev	TGGAGGCCTCTCCAGAAT	Quantitative PCR
Mef2a_fw	CACGCATAATGGATGAGAGGAA	Quantitative PCR
Mef2a_rev	CAGAGCACACTGAGTTCATAGG	Quantitative PCR
Mef2c	AGGACAAGGAATGGGAGGATA	Quantitative PCR
Mef2c_rev	TGTTGAAGCCAGACAGAGATG	Quantitative PCR
Beta-actine_fw	CCTAGGCACCAGGGTGTGAT	Quantitative PCR
Beta-actine_rev	GCCTCGTCACCCACATAGGA	Quantitative PCR
sgRNA	CGGCTCCAAAAGTACTTTGA	CRISPR-READI Mymx-T2A-Cre
Cre_fw	AGGTGTAGAGAAGGCACTTAGC	Genotyping transgene Mymx-T2A-Cre
Cre_rev	TCAATCGCCATCTTCCAGCAGG	Genotyping transgene Mymx-T2A-Cre

Development • Supplementary information

5'junction_fw	CGTGCCTGAGGTACAGTCTAA	Genotyping 5' junction Mymx-T2A-Cre
5'junction_rev	TCAATCGCCATCTTCCAGCAGG	Genotyping 5' junction Mymx-T2A-Cre
3'junction_fw	AGGTGTAGAGAAGGCACTTAGC	Genotyping 3' junction Mymx-T2A-Cre
3'junction_rev	GTAGACGGGACAGTTTGGATTTA	Genotyping 3' junction Mymx-T2A-Cre

Development • Supplementary information
